# Supplementary material for: Improving the diagnostic yield of exome- sequencing by predicting gene–phenotype associations using large-scale gene expression analysis
Source: Nat Commun. 2019 Jun 28;10:2837. doi: 10.1038/s41467-019-10649-4 (PMC6599066; doi:10.1038/s41467-019-10649-4)
Supplement: Supplementary file 1 — Supplementary Information [file 41467_2019_10649_MOESM1_ESM.pdf]

Improving the diagnostic yield of exome-sequencing by predicting disease symptoms using large-scale gene expression analysis

Patrick Deelen, Sipko van Dam, Johanna C. Herkert, Juha M. Karjalainen, Harm Brugge, Kristin M. Abbott, Cleo C. van Diemen, Paul A. van der Zwaag, Erica H. Gerkes, Evelien Zonneveld-Huijssoon, Jelkje J. Boer-Bergsma, Pytrik Folkertsma, Tessa Gillett, K. Joeri van der Velde, Roan Kanninga, Peter C. van den Akker, Sabrina Z. Jan, Edgar T. Hoorntje, Wouter P. te Rijdt, Yvonne J. Vos, Jan D.H. Jongbloed, Conny M.A. van Ravenswaaij-Arts, Richard Sinke, Birgit Sikkema-Raddatz, Wilhelmina S. Kerstjens-Frederikse, Morris A. Swertz, Lude Franke

Supplemental Notes **3**

Supplementary note 1: Processing and quality control of public RNA-seq data................3

Supplementary note 2: Using alternative tools to prioritize the candidate genes found using GADO 7

Supplementary note 3: GeneNetwork website 8

Supplementary Figures **10**

Supplementary figure 1: Selection of parent HPO term if GADO does not have significant predictive power for query term. 10

Supplementary figure 2: Performance of disease gene prioritization compared to random permutation..11

Supplementary figure 3: The prioritization Z-score when using a maximum of 5 random HPO terms to predict known diseases genes are strongly correlated to using all annotated HPO terms. 12

Supplementary figure 4: Correlation between the GADO prioritization Z-scores and the ExAC missense constraint. 13

Supplementary figure 5: Comparison of GADO performance with the level of evidence for each cardiomyopathy-related gene. 14

Supplementary figure 6: Including 10% random genes when predicting HPO-terms has a marginal effect on prediction accuracy 15

Supplementary figure 7: Rank of the known causative gene among the candidate disease causing variants. 16

Supplementary figure 8: Correcting for biases in co-expression networks. 17

Supplementary figure 9: Histogram of the gene types included in our analyses. 19

Supplementary figure 10: PCA plot of 36,761 samples. 20

Supplementary figure 11: Investigation of principal components capturing technical biases. 21

Supplementary figure 12: Variance explained by first 1,588 PCs. 23

Supplementary figure 13: Visualization of PC1 to PC 10 of PCA over gene correlation matrix. 24

Supplementary figure 14: Outlier genes in PC 8 and PC 9 of PCA over gene correlation matrix. 26

Supplementary figure 15: PC sample scores to distinguish different tissues. 27

Supplementary figure 16: Outlier samples in PC sample scores of PC 8 and PC 9........... 29

Supplementary tables 30

Supplementary table 1: A list of 83 diagnosed patients with Mendelian disorders and corresponding predictions with GADO.. 30

Supplementary table 2: Comparison between GADO and Exomiser predictions using a list of 83 diagnosed patients with Mendelian disorders. 36

Supplementary table 3: A list of 61 undiagnosed patients with suspected Mendelian disorders. 40

Supplementary References 43

Supplemental Notes

Supplementary note 1: Processing and quality control of public RNA-seq data

All RNA-seq data used in this project was acquired from the European Nucleotide Archive (ENA) database [1]. Of the 67,090 human RNA-seq samples, with at least 500,000 reads, registered in the ENA on June 30, 2016 (supplementary data 1), 67,019 were successfully downloaded. For 71 of the registered samples, the files were missing. Sample annotations were acquired from [2,3] and through manual curation based on study meta-information in the ENA database (supplementary data 1).

Gene expression quantification

The 67,019 downloaded samples were mapped to transcript annotations from Ensemble release 83 which uses build GRCh38.p5 of the human genome [4] using Kallisto [5] version 0.42.4, and the number of reads assessed. The number of reads mapped per sample was obtained from the Kallisto summary file. The following genome files were used:
ftp://ftp.ensembl.org/pub/release-83/fasta/homo_sapiens/cdna/Homo_sapiens.GRCh38.cdna.all.fa.gz
ftp://ftp.ensembl.org/pub/release-83/fasta/homo_sapiens/ncrna/Homo_sapiens.GRCh38.ncrna.fa.gz
These files were merged and used to build the Kallisto reference index file. The following setting, in addition to all default settings, was used: –k 31.

The following Kallisto settings were used mapping all 67,019 samples using default settings for paired-end data mapping. For single-end data mapping we used the following settings in addition to the defaults: –l 200 and –s 20 –bias.

After obtaining the transcript counts per sample, these transcript-level counts were summed to gene-level counts for each sample of which we took the log2.

Gene quality control

We quantified 66,233 genes, which were filtered on the criteria described below, after which 56,435 genes remained. Twenty-nine gene names were duplicates/identical. After these were removed, 66,203 genes remained. Of these, 3,628 genes are not expressed (0 reads detected among 31,499 samples) and were removed, leaving 62,575 genes. Next, we detected a number of duplicate genes (100% sequence similarity). Since these genes with perfect sequence similarity have exactly the same number of reads mapping, we were concerned they would appear as perfectly co-expressed genes in our analysis. Most of these genes are either incorrectly mapped genes in the genome build or duplicates of their

biological counterpart. Due to their high sequence similarity they are indistinguishable to the mapping tool (potentially introducing false correlations). To avoid potential biases resulting in deceptively high co-expression values, we decided to remove this bias prior to our analysis. 5,471 of these were not located on chromosomes (but on scaffolds), and were removed, leaving 57,104 genes. Another 665 genes had identical transcripts: different IDs, but 100% identical sequences (e.g. ENST00000442165 and ENST00000446969).
An additional four genes had no expression in any of the remaining samples after removing outlier/poor-quality samples, as described below, and were also removed prior to the PCA analysis. The 56,435 genes that remained were used for our analyses (supplementary figure 9).

Sample quality control
We excluded all samples in which less than 70% of the reads successfully mapped to the genome, as reported by Kallisto, resulting in 36,761 samples.

Principal component analysis to identify outlier samples
To identify outlier samples, we conducted a principal component analysis (PCA) along the following steps. First, all estimated counts were log2 transformed. Second, the data was quantile normalized. Third, the covariance over the samples was calculated. Fourth, genes without variance were removed from the dataset. Fifth, a PCA was conducted on the covariance matrix. An arbitrary cut-off on PC 1 was selected at 0.0049 (supplementary figure 10), leaving us with 32,142 samples.

Removal of non-Illumina samples
Since only a small number of samples that passed quality control (147 samples, <0.5% of the total number of samples) were not sequenced on Illumina machines, we removed these to avoid potential biases as a result of these different sequencing tools. This left 31,995 samples in our dataset.

Removing duplicate samples
A number of samples had identical values for all genes. Upon inspection, some of these samples appeared to be have been used by multiple studies and uploaded to the ENA database multiple times. To remove duplicate samples, we identified all samples with a correlation >0.9999, randomly selected one of them to include and removed the other. After this step, 31,499 samples remained.

Removal of technical biases

The remaining samples were normalized using DeSEQ [6]. To identify potential technical biases in our data, we calculated the correlation between the PC-scores for each PC and the following potential confounders: read length, paired/single end, total reads in the dataset and percentage mapping reads (supplementary figure 11). We found that all these factors significantly correlated to our sample PC scores for multiple PCs (p-value < 0.01), indicating that these technical factors would affect the co-expression detected in the dataset, if not removed. We decided not to correct for GC content per gene as this may also have biological meaning [7]. For a manual of the covariate removal pipeline we refer to: https://github.com/molgenis/systemsgenetics/tree/master/eqtl-mapping-pipeline. To remove covariates, we used the "adjustcovariates" option.

Principal component analysis

After correcting our dataset for technical biases, we conducted the following steps on the matrix. First, we calculated the correlation over the genes. Second, we conducted a PCA over the correlation matrix over the genes. Third, we calculated PC scores for each sample for all PCs.

After the quality control steps described above, we conducted a co-regulation analysis using the 31,499 sample by 56,435 gene matrix. The co-regulation analysis was performed using the PC eigencoefficients of the genes for each of the reliable PCs obtained from our gene-co-expression matrix. To determine which PCs are reliable, Cronbach's alpha [8] was calculated for each PC (based on PCA of the gene-correlation matrix). Those PCs with a Cronbach's Alpha ≥ 0.7 were considered reliable, and is a commonly used cutoff [9]. In total, 1,588 PCs have a Cronbach's Alpha ≥ 0.7. Additionally, we calculated the variance explained by each of these PCs and found the first 1,588 PCs explain 66 percent of the variance (supplementary figure 12). By including signals from only these PCs, we aimed to remove signals that are not reliable from our analysis. This method was previously shown to perform better than using the correlation matrix directly [10].

Inspection of gene PC eigencoefficients

To investigate if any technical biases were present for the different gene types (coding, miRNA, pseudogene, etc.), we plotted the gene eigencoefficients for the first 10 PCs and colored the genes by biotype (supplementary figure 13) and detected an outlier cluster on PC8 and PC9, which were further investigated (supplementary figure 14).

Inspection of sample PC scores

To better understand the origin of the outlier genes in the eigenvector coefficients of PC 8 and PC 9, we investigated the PC scores of the samples for these PCs. Additionally, we created a plot for each of the sample PC scores of the first 10 PCs (supplementary figure 15). We observed that there is a clear biological explanation for these outliers, and therefore we decided to retain these signals in the data (supplementary figure 16).

Data visualization of sample PC scores using a t-SNE plot

To identify clusters for each cell type and tissue type, we used the sample PC scores, which indicate how strong the signal of each sample is for each PC in the data. Here, each PC is a gene expression signature for the complete set of genes. To visualize how the samples cluster in a two dimensional figure, we constructed a t-SNE plot [11] based on these sample PC-scores using the Rtsne library [12] (version 0.13). The t-SNE was run with a perplexity of 50, and we ran 10,000 iterations on our sample PC score matrix. We found that single clusters were visible for many cell- and tissue-types (**Figure 2**a). Most of these clusters contain samples from different studies, which suggests that these clusters are not merely a representation of study-specific biases. The fact that studies with multiple cell/tissue types show multiple clusters further supports the suggestion that the clusters are not driven by non-biological inter-study differences.

Supplementary note 2: Using alternative tools to prioritize the candidate genes found using GADO

We attempted to prioritize the candidate genes we identified in our unsolved cases using the following existing tools: Exomiser, ENDEAVOR [13], ToppGene [14].

Exomiser
We used the same version as in Supplementary methods 2 and used the default settings. We sorted the results based on the "EXOMISER_GENE_COMBINED_SCORE".

ToppGene
There is no option in ToppGene to combine the results of multiple HPO terms and we therefor only applied ToppGene to the cases with a single HPO term listed. Since ToppGene does not work with HPO terms directly we extracted a list of gene names from the used HPO term from the HPO database we downloaded.

ENDEAVOR
Similair to ToppGene, ENDEAVOR does not work with HPO terms directly and does not provide an option to integrate multiple prioritizations. We therefor used the same samples and extracted gene-lists as for ToppGene. With the added limitation that the maximum number of supported genes in the training data was 200, if more than 200 genes were associated to an HPO term we selected a random subset. The number of genes that can be ranked is also limited to 200, for the cases for which GAVIN selected more than 200 genes we only ranked a random subset of genes while making sure that the gene GADO identified was present within this subset.

Supplementary note 3: GeneNetwork website

We implemented the following functionality for www.genenetwork.nl.

GADO gene prioritization
Prioritize potential causative disease genes for patients based on HPO terms or a group of genes annotated to a patient, the GADO tool will rank all genes based on how likely they are to be related to the patient's phenotype. These can be further filtered for genes of interest, by providing a list of genes known to harbor candidate causative variants.

We also visualize the relations between the provided HPO terms using a heatmap. This heatmap is created by correlation the prioritization Z-score of two HPO terms.

Gene function predictions
Per gene we have made the prediction for the GO, Reactome, KEGG gene sets and HPO terms can be retrieved.

Gene-gene co-regulation
The gene co-regulation scores were calculated by correlating the eigencoefficients of each gene pair after the eigencoefficients were standard normalized per gene, followed by a standard normalization per PC. This is done so each PC weighed equally when determining the co-regulation between two genes [10]. The p-values of co-regulated genes can be queried via the website.

Gene network visualization
Edges are drawn between two genes/nodes based on the co-regulation z-score. The cutoff at which a line/edge between two genes should be drawn can be manually altered with the bar in the top right corner. The network is drawn based on a force directed layout and clusters are assigned using affinity propagation [15].

HPO, Reactome, KEGG and GO enrichment calculations
On the network page it is possible to retrieve which HPO, Reactome, KEGG and GO categories are enriched among the visualized genes. It is also possible to retrieve this for a sub-selection of these genes. The enrichment is calculated based on the z-scores of each of these genes for each category. For each category/term, a Mann-Whitney U test is conducted between the z-scores of the genes in the network versus the z-scores of genes that are not part of the visualized network. The pathways with the most significant p-values are then ranked highest.

It is also possible to identify which other genes are strongly co-regulated with those visualized in the network. This is done similarly to how the correlation between a gene and a pathway is calculated, as described above in “Gene function and HPO association predictions”. First, the z-scores for each PC of the genes visualized in the network is calculated. After the z-scores of this group of genes have been calculated for each pathway, the correlation of the PC coefficients for each gene not in the network with these z-scores is calculated. The genes with the most significant correlation are ranked highest.

Supplementary Figures

Supplementary figure 1: Selection of parent HPO term if GADO does not have significant predictive power for query term.

If the predictive power for a particular query HPO term is not significant (poor performance), the parent terms are instead suggested to make predictions. If one of the parent terms also does not have significant predictive power, then its parents are suggested. The algorithm progresses up the HPO tree until alternative terms are found for which GADO does have significant predictive power. The example shown is for HP:0002037 (Inflammation of the large intestine).

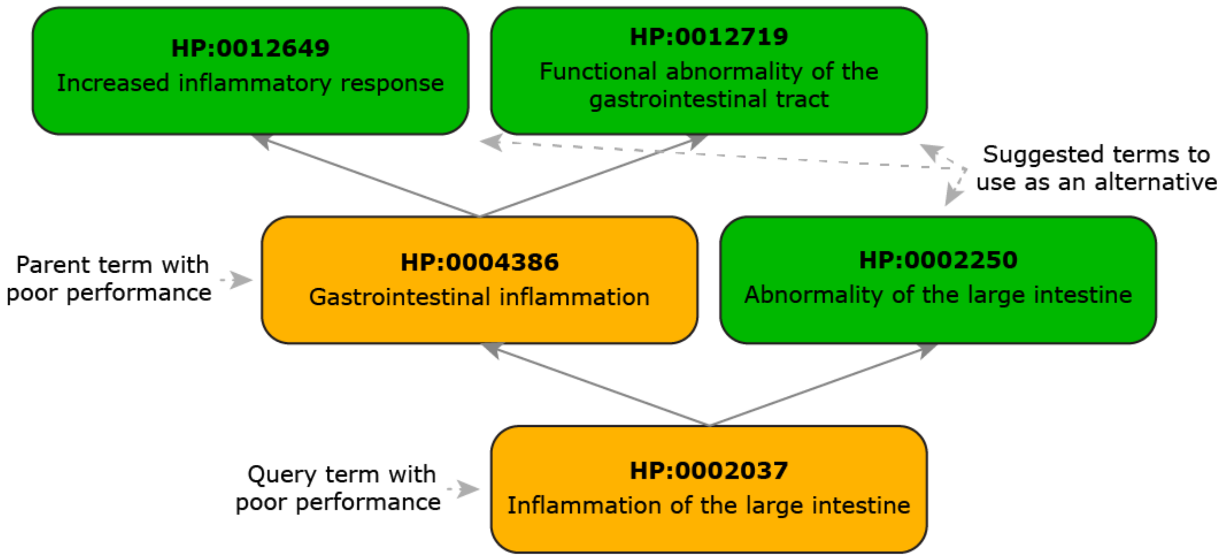

Supplementary figure 2: Performance of disease gene prioritization compared to random permutation.

(a) OMIM disease genes and provisional disease genes have significantly stronger z-scores compared to permuted disease genes (T-test p-values: 2.16×10-532 & 5.38×10-80, respectively). We also observe that the predictions of the provisional OMIM genes are, on average, weaker than the other OMIM disease genes (T-test p-value: 1.89×10-7).

(b) Ranking the disease based on z-scores shows GADO's ability to prioritize the causative gene for a disease among all OMIM genes. For 49% of the disorders the causative gene is ranked in the top 5%.

(c) We observe a clear relation between the prioritization z-scores and the gene prioritization *Z*-scores (Pearson r = 0.54). We don't observe this relation in the permuted results.

(d) GeneNetwork performs best for genes with high predictability scores.

(e) The different groups have similar distributions of gene predictability scores.

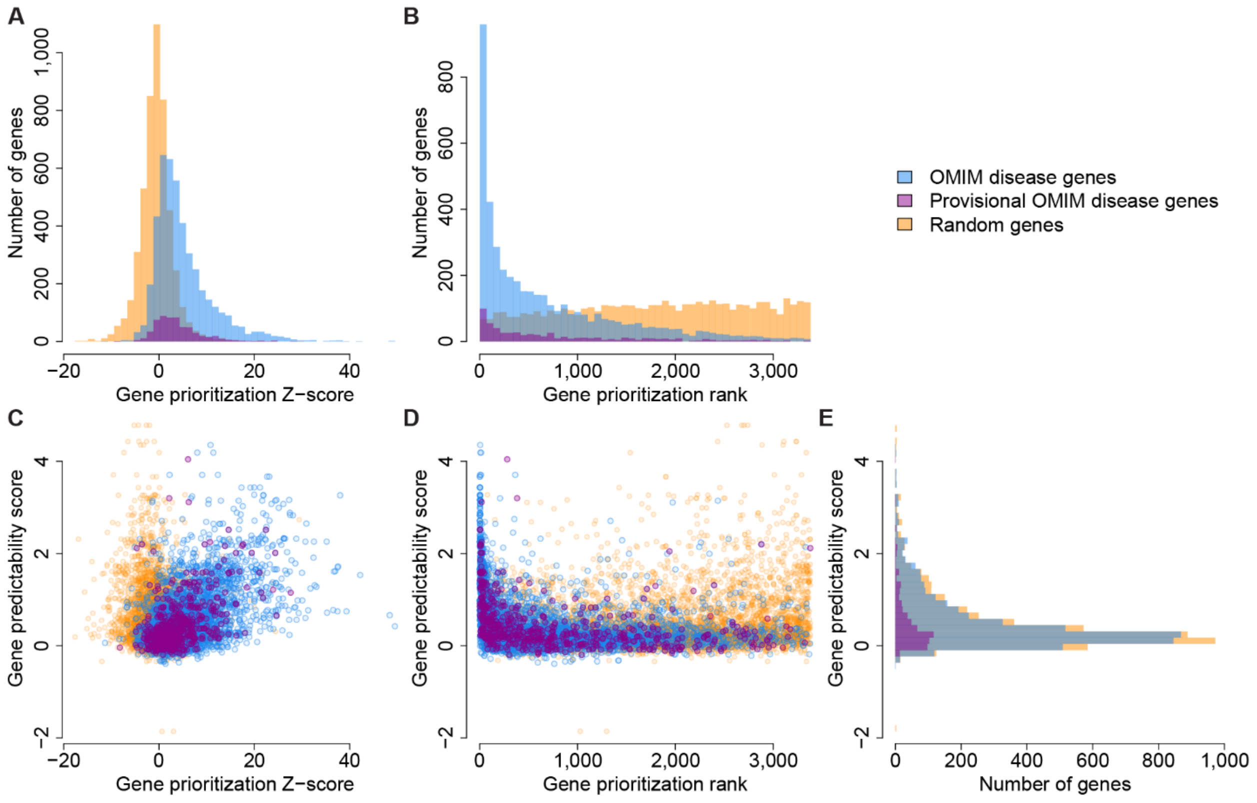

Supplementary figure 3: The prioritization Z-score when using a maximum of 5 random HPO terms to predict known diseases genes are strongly correlated to using all annotated HPO terms.

The Pearson correlation between the prioritization Z-scores is 0.86. While this indicates that GADO also works well when using only 5 HPO terms, we believe this is an underestimate, since we randomly select 5 of the annotated HPO terms per disease. We expect that in reality clinicians usually will try to enter HPO terms that describe clearly different phenotypes, yielding more informative results.

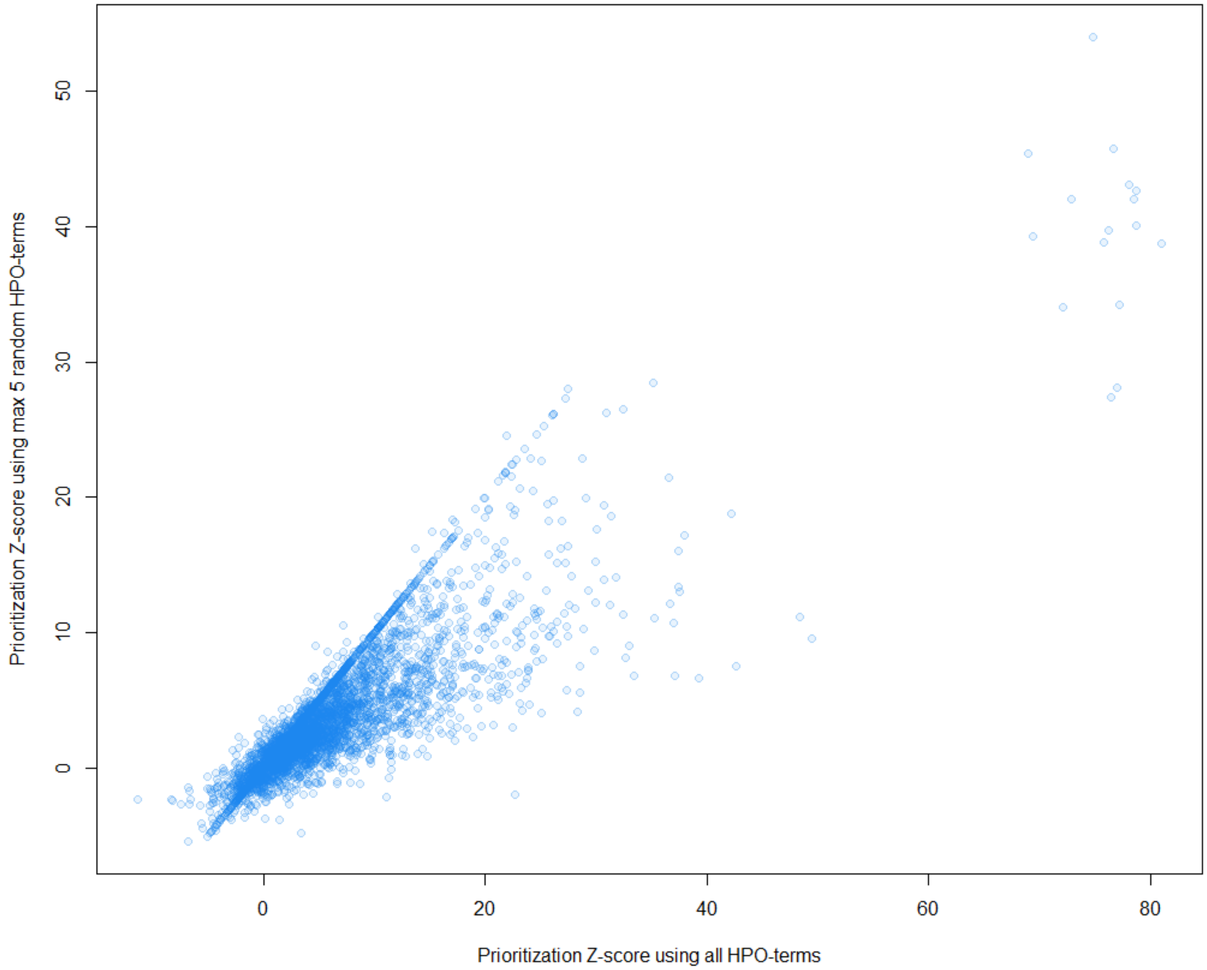

Supplementary figure 4: Correlation between the GADO prioritization Z-scores and the ExAC missense constraint.

(a) The correlation between the ExAC missense constraint score and the number of submission to Clinvar is detected.

(b) The correlation between the ExAC missense constraint score and the GADO gene prioritization Z-scores is not observed.

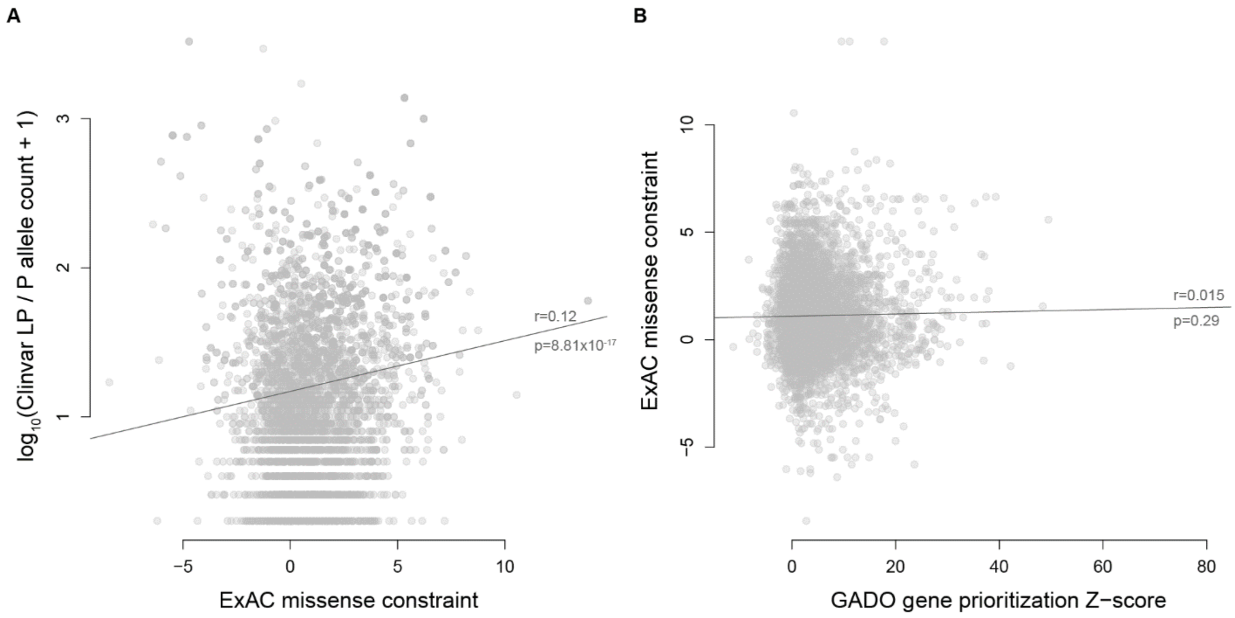

Supplementary figure 5: Comparison of GADO performance with the level of evidence for each cardiomyopathy-related gene.

All genes annotated to the HPO term 'cardiomyopathy' (HP:0001638) supplemented with genes recently reviewed in literature [16,17], were given a score based on the level of supporting evidence in literature suggesting each of these genes is involved in cardiomyopathy. The genes were scored independently by two clinicians based on the number of publications available, segregation of a given variant and functional evidence. In case a gene was scored differently, the papers were full-read and discussed until consensus was reached. Genes with much evidence tend to have higher gene prioritization Z-scores and higher gene predictability scores. We observed that GADO poorly ranks genes that cause disease through secondary effects. For example, the *TTR* gene has a low prioritization Z-score but a high predictability score, even though this gene is known to play a role in cardiomyopathy.

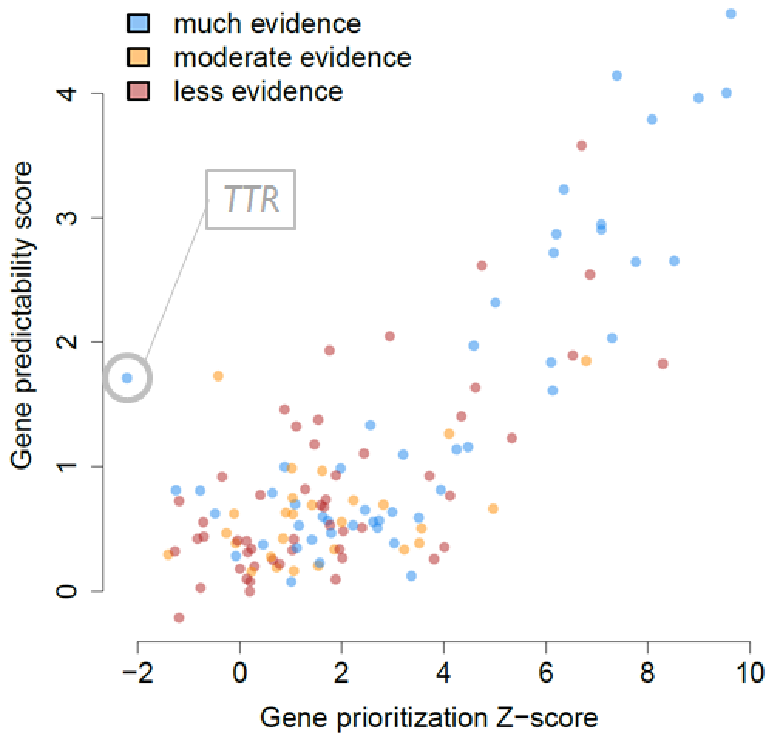

Supplementary figure 6: Including 10% random genes when predicting HPO-terms has a marginal effect on prediction accuracy

To ascertain the effect of false positive disease gene-associations we randomly added 10% genes to each HPO term and recalculated predictions and AUC’s. The AUC’s when including the random spike-in was strongly correlated to the original AUC (r: 0.97). The median AUC dropped slightly from 0.73 to 0.71.

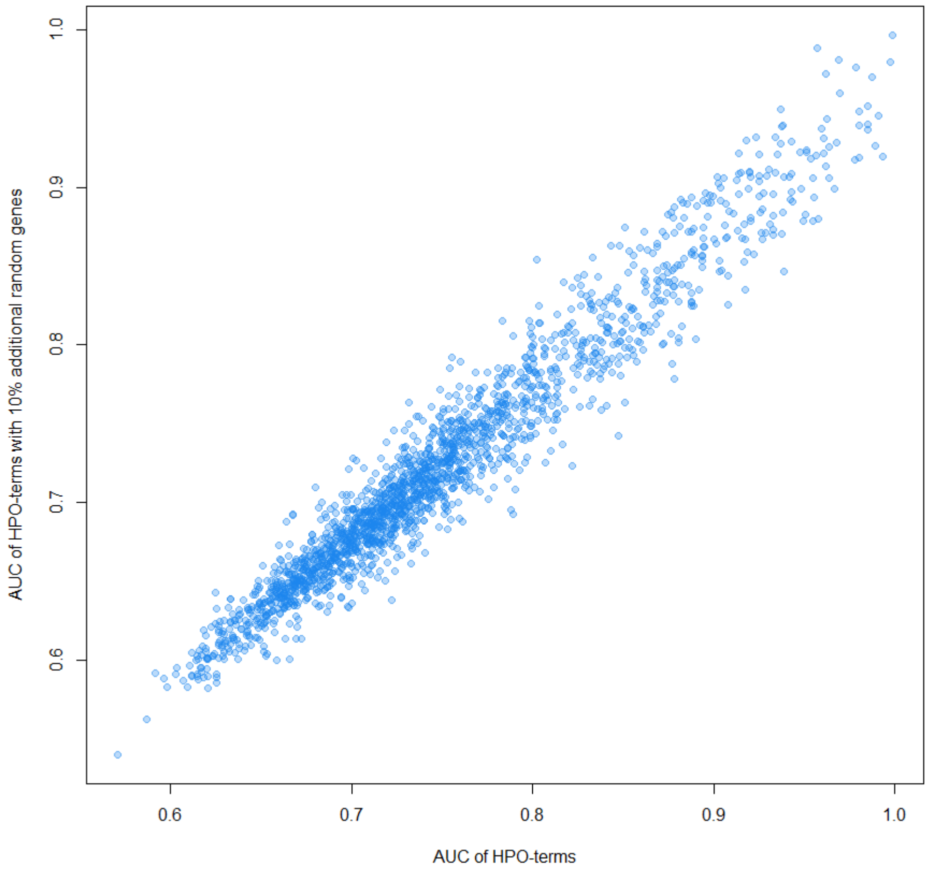

Supplementary figure 7: Rank of the known causative gene among the candidate disease causing variants.

Exome sequencing data of 83 patients with a known genetic diagnosis were used. Their phenotypic features, as listed in their medical records prior to the genetic diagnosis, were used. On average, per patient, GADO yielded 56 possible disease-causing genes with variants that are rare and predicted to be deleterious.

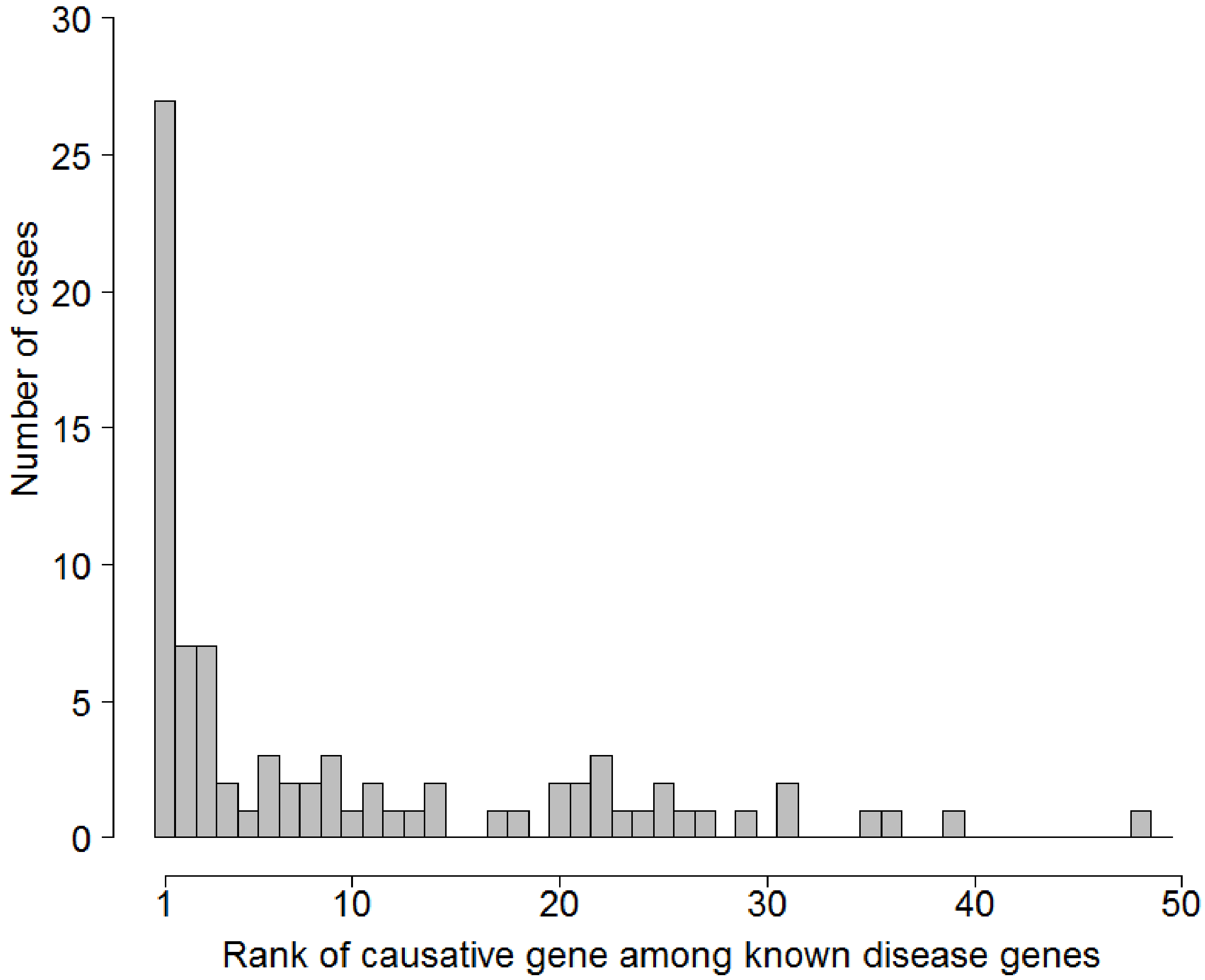

Supplementary figure 8: Correcting for biases in co-expression networks.

(a) One common problem with co-expression analyses is its scale-free properties [10]: when using a certain co-expression correlation threshold to declare an interaction, the topology of the network becomes such that for the majority of genes (so-called spoke genes) very few significant co-expression relationships are found, whereas for a very limited number of genes (so-called hub genes) many interactions are found [18]. We observed this in our dataset as well: first of all, when using a Pearson correlation threshold of at least 0.3, we observed that the distribution of number of interactions per gene showed a power-law distribution, confirming the scale-free topology of this network (r^2=0.76). For instance, we identified 16,797 genes that each had less than 10 co-expression interactions but 17,320 genes that each had at least 1,000 interactions. This has ramifications for how HPO functions can be predicted: if we, for instance, would study a gene that currently lacks any HPO annotation and we would like to predict HPO terms, we could, for instance, assign HPO terms from genes that are strongly co-expressed with that gene. However, in 1 of 20 cases that gene is co-expressed with a hub gene that has 1,000 interactions in 1/20 cases. Phrased differently, the known HPO terms of this hub gene will be assigned to 1,000 other genes as well.

(b) To overcome this, we decomposed the co-expression matrix into individual principal components, and for the prediction of HPO terms we weigh each of these components. As a consequence, GADO is able to make HPO inferences for the majority of protein-coding genes. For 10,318 genes, at least one HPO term is predicted with a prioritization z-score ≥ 5. Additionally, we observed that hub genes had not been assigned more HPO terms than spoke genes, indicating that our HPO predictions are not driven by the topology of co-expression networks (r^2 = 0. 013).

(c) This also alleviates strong biases that exist in literature towards well studied genes such as *TP53*, *TNF*, *EGFR*, *VEGFA* and *APOE* (each studied in over 40,000 papers [19]), whereas nearly half of the protein-coding genes have rarely been studied, and thus have not yet received HPO annotation. This is also reflected in the high-quality interactions reported by STRING. Here we also observed a scale free network topology among the high-quality (score ≥ 0.7, this is the definition used by Exomiser) interactions which will bias HPO term assignment based on STRING interactions to well-studied genes (power law fit r^2 = 0.87). Well studied genes contain more interactions and are therefore more likely to be assigned to an HPO term.

(d) While most interactions in the STRING database are based, at least partially, on existing knowledge, STRING does contain some high-quality interaction solely based on co-

expression. In principle this allows Exomiser to assign HPO terms to genes without any prior annotation. However only 1,244 human genes have at least one such high-quality interaction and, since co-expression networks have a scale free topology, we also observed that the number of interactions per gene follows a power-law distribution (r^2 = 0.64).

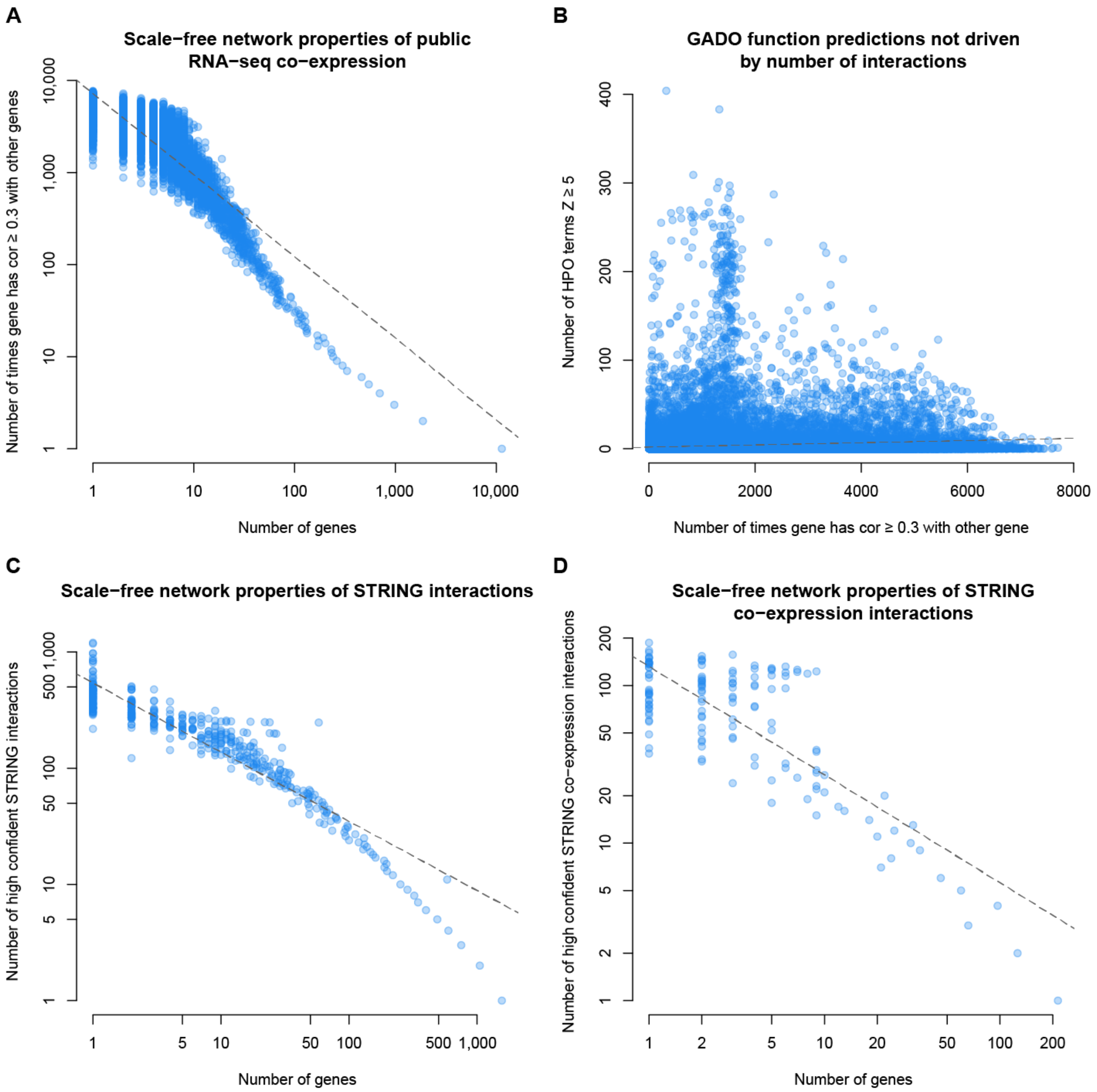

Supplementary figure 9: Histogram of the gene types included in our analyses.

Gene type annotations were obtained from Ensembl build 38, version 83 [4]. Most prevalent gene type bars are colored in accordance with supplementary figures 8 and 9.

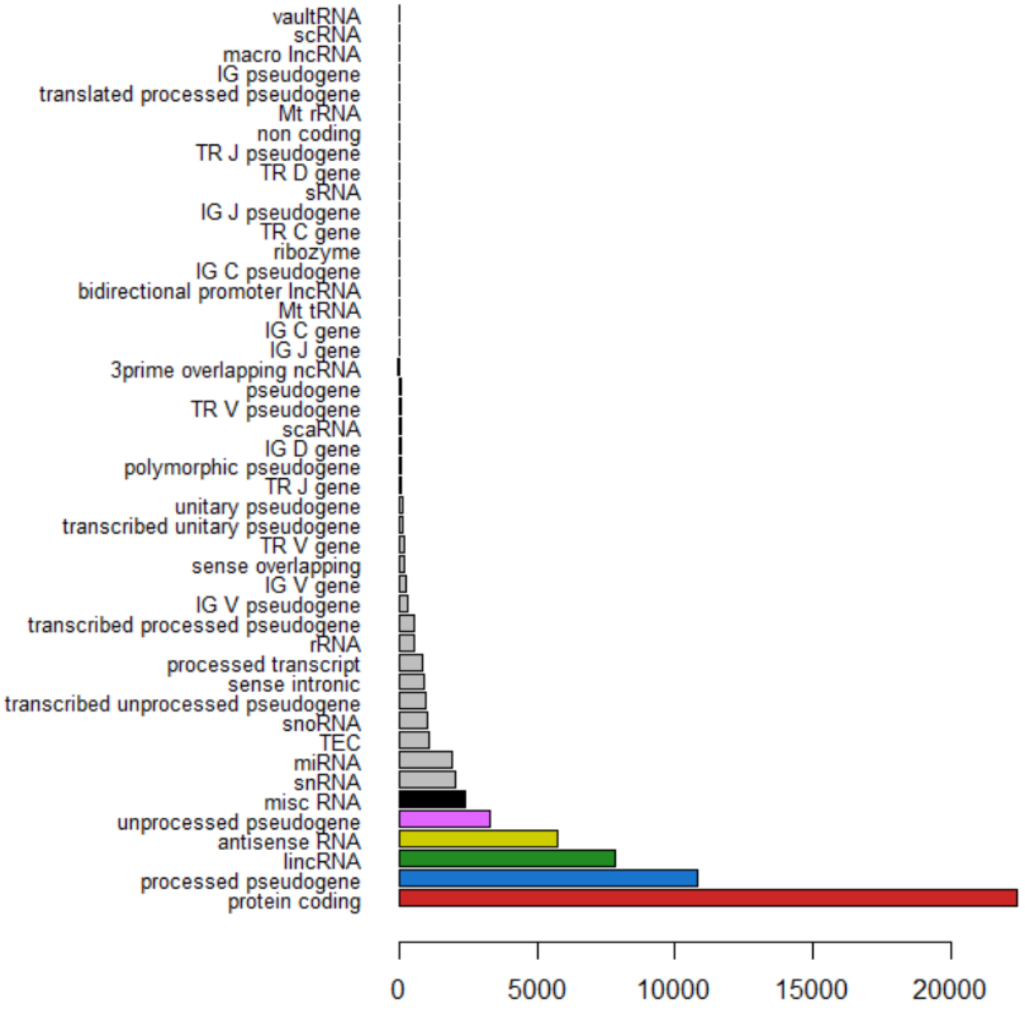

Supplementary figure 10: PCA plot of 36,761 samples.

Each dot represents a sample. Annotated samples are plotted on top and annotations are retrieved from supplementary data 1. Cutoff was arbitrarily set at 0.0049 to retain 32,142 samples, retaining the largest cluster of samples while removing the outlier clusters and all samples with a similar signal for PC1.

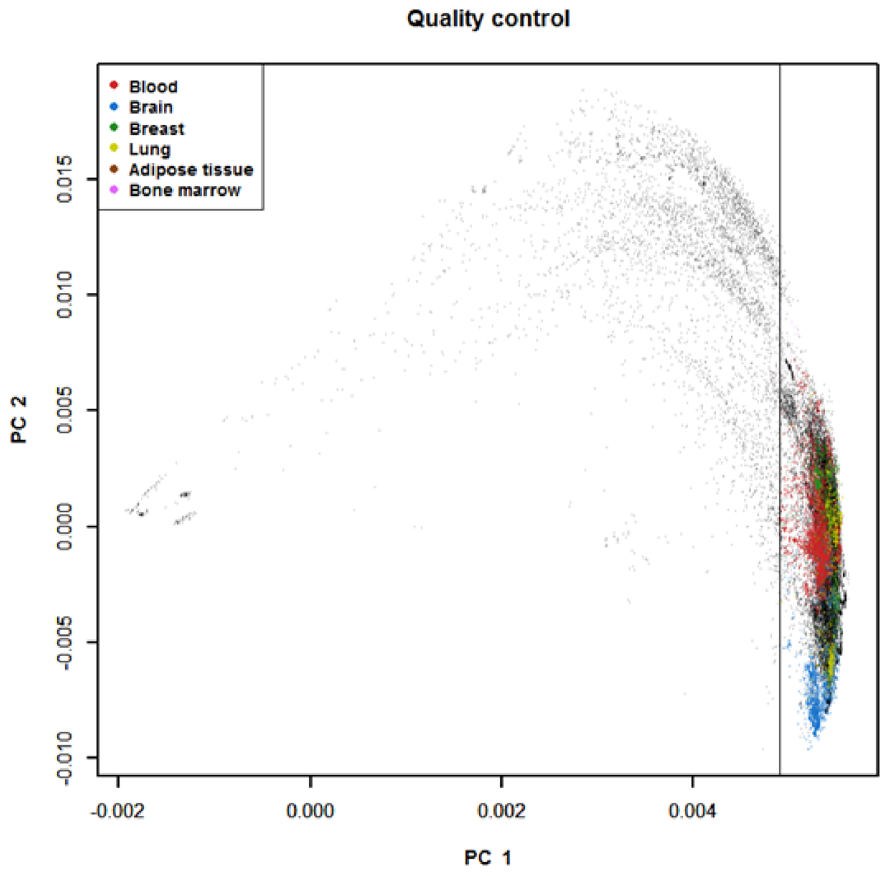

Supplementary figure 11: Investigation of principal components capturing technical biases.

We determined the correlation between read length, total number of reads in the dataset and the percentage mapped. This was determined for the 307 PCs with a Cronbach's Alpha > 0.7. Similarly, we conducted a Wilcoxon test between the PC scores for the single end and paired end samples and converted these to z-scores. All z-scores lower than -38.53 (p-value < 1.98 x 10^{-323}) are reported as -38.53. For each of the four statistics (read length, total reads in dataset, percentage mapped reads and single/paired end), we assessed the bias in all significant PCs (left) and selected the one with the largest bias for visualization (right).

We found that all of these factors were significantly correlated to our PC scores for PCs (p-value < 0.01), indicating that these technical factors would affect our co-expression results if not removed.

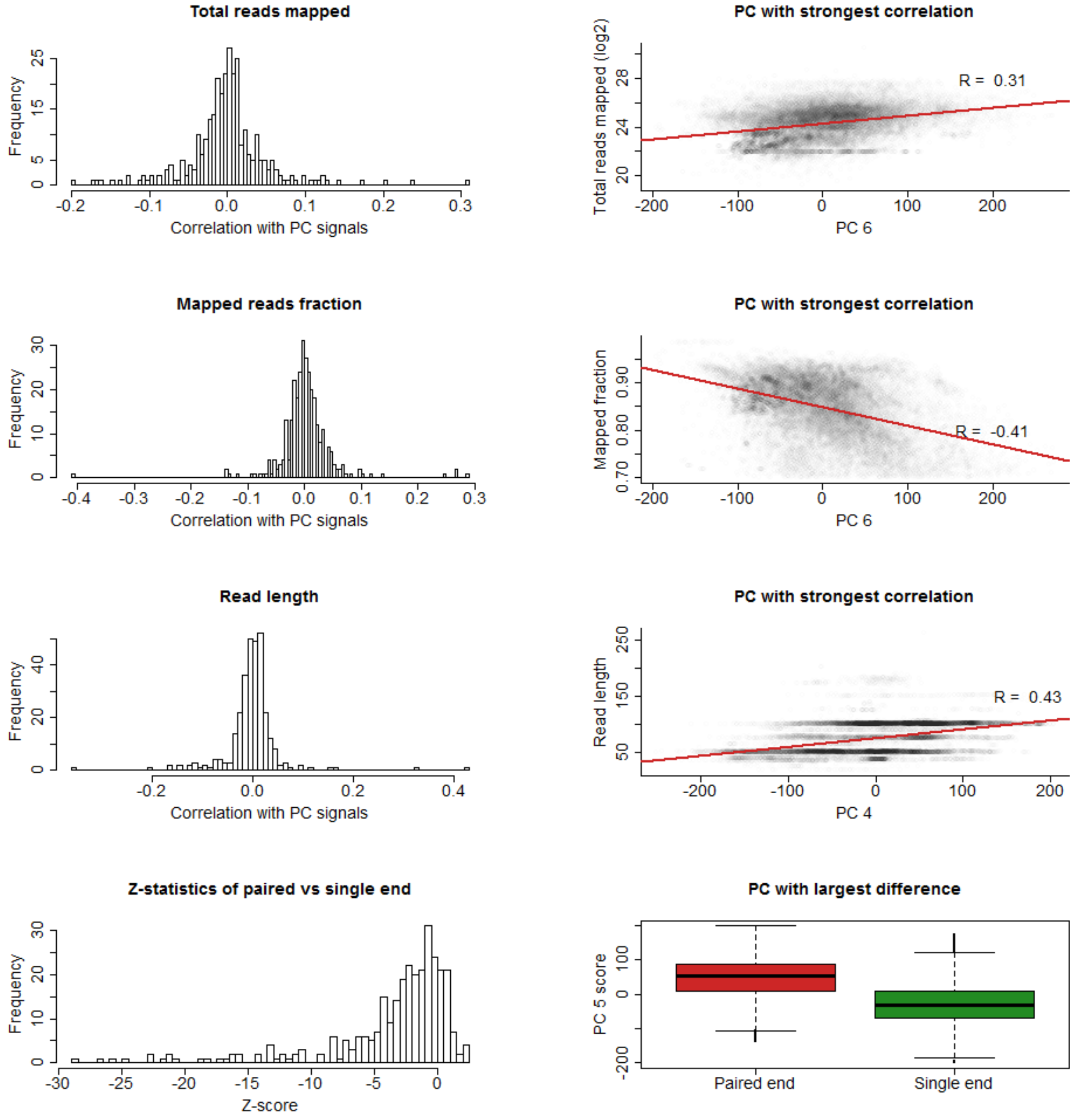

Total reads mapped
Frequency
Correlation with PC signals
PC with strongest correlation
Total reads mapped (log2)
R = 0.31
PC 6
Mapped reads fraction
Frequency
Correlation with PC signals
PC with strongest correlation
Mapped fraction
R = -0.41
PC 6
Read length
Frequency
Correlation with PC signals
PC with strongest correlation
Read length
R = 0.43
PC 4
Z-statistics of paired vs single end
Frequency
Z-score
PC with largest difference
PC 5 score
Paired end
Single end

Supplementary figure 12: Variance explained by first 1,588 PCs.

The first 100 PCs together explain 46% of the variance. The first 1,588 PCs together explain 66% of the variance together.

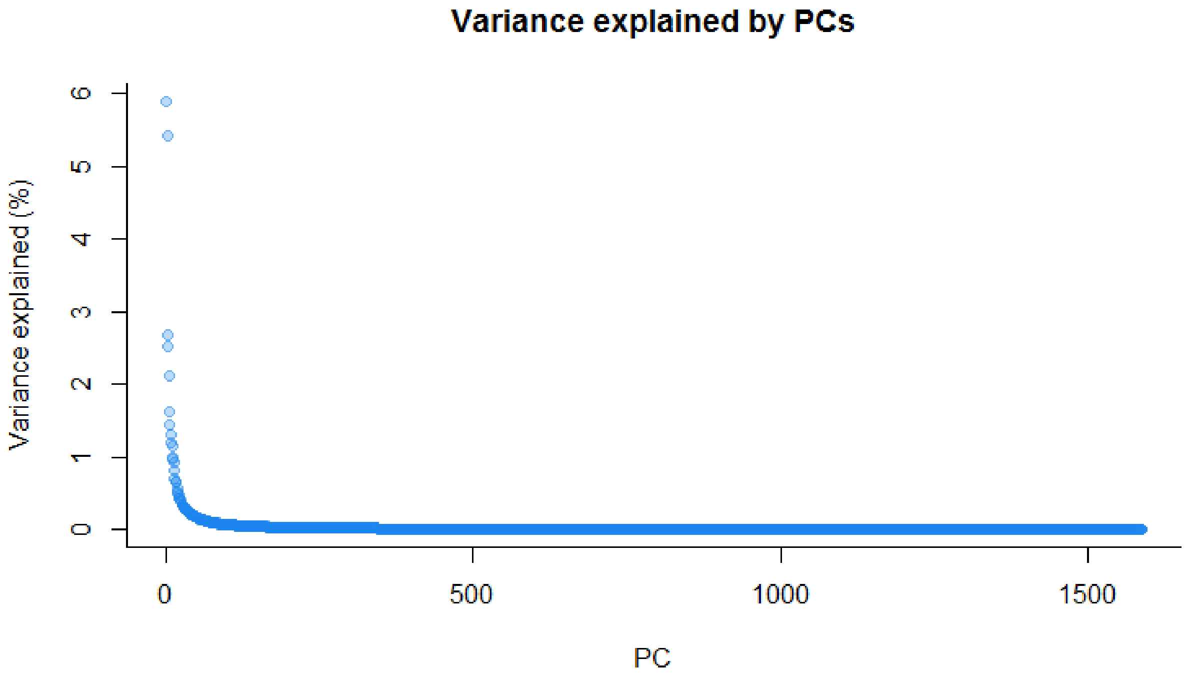

Supplementary figure 13: Visualization of PC1 to PC 10 of PCA over gene correlation matrix.

To identify any potential biases remaining in the data, the first 10 PCs were investigated for outlier patterns. A clear group of outliers was identified in PC7 and PC8, which was further investigated.

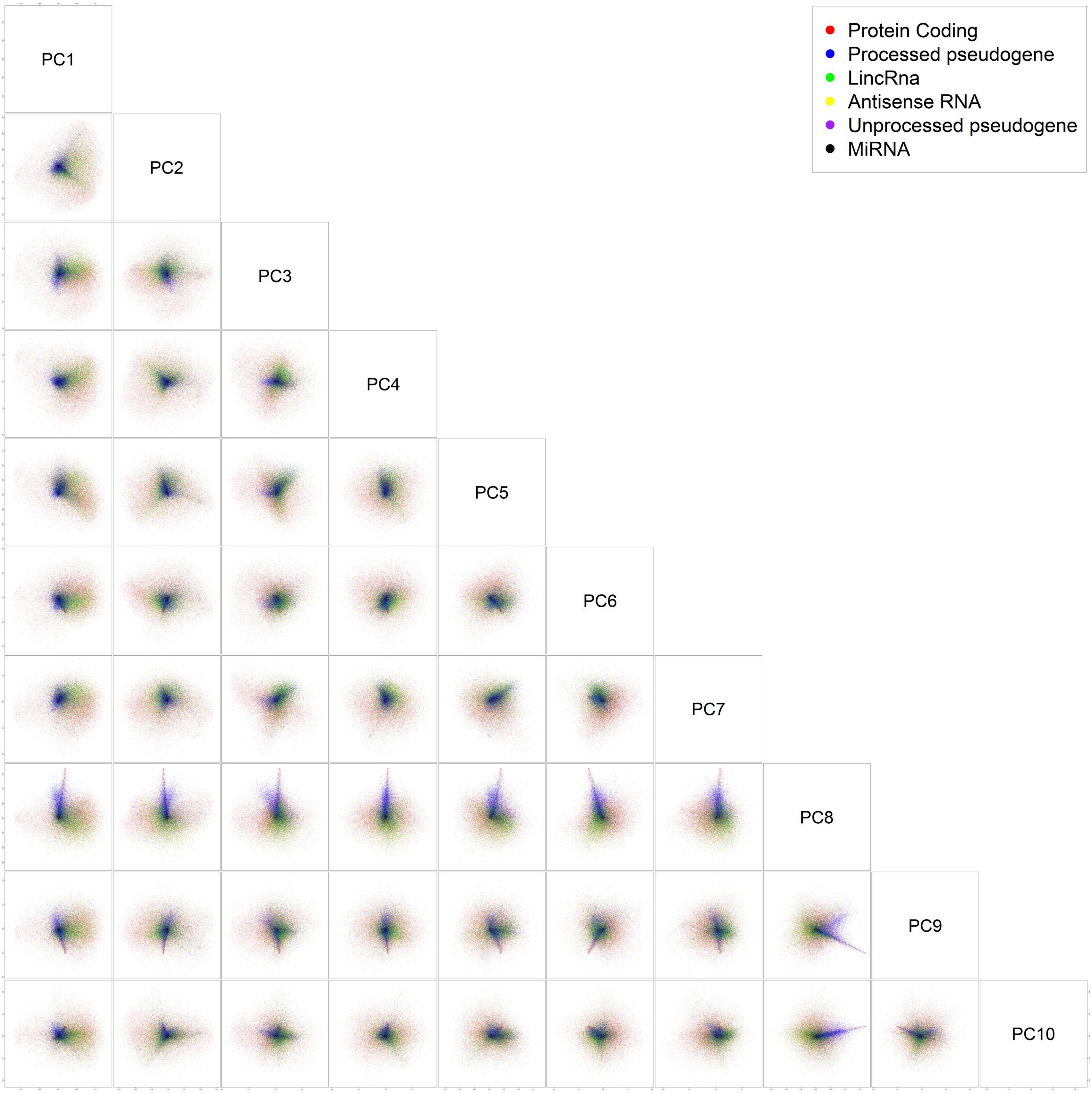

Protein Coding
Processed pseudogene
LincRna
Antisense RNA
Unprocessed pseudogene
MiRNA
PC1
PC2
PC3
PC4
PC5
PC6
PC7
PC8
PC9
PC10

Supplementary figure 14: Outlier genes in PC 8 and PC 9 of PCA over gene correlation matrix.

Arbitrary cutoffs to select outlier genes for functional enrichment analysis were set at PC8 > 0.010 and PC9 < -0.005. Using gene function enrichment analysis, we found these genes to be enriched for Olfactory Receptor pathway genes (p-value = 2.980E-276), as determined using the ToppFun functional enrichment analysis feature [14].

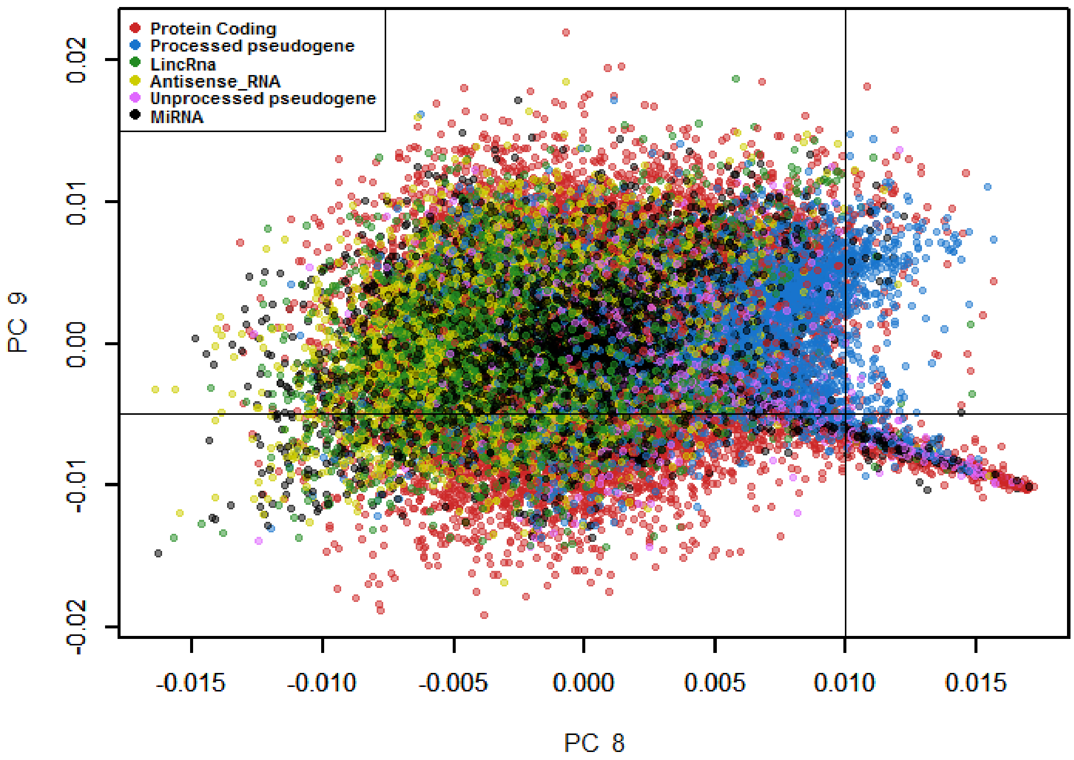

Supplementary figure 15: PC sample scores to distinguish different tissues.

To determine if the first 10 PCs can distinguish samples originating from different tissues, we plotted the PC scores of each pair. Tissues for which at least 500 samples are annotated and colored. The outlier samples in PC 8 and PC 9 were investigated in more detail (supplementary figure 10).

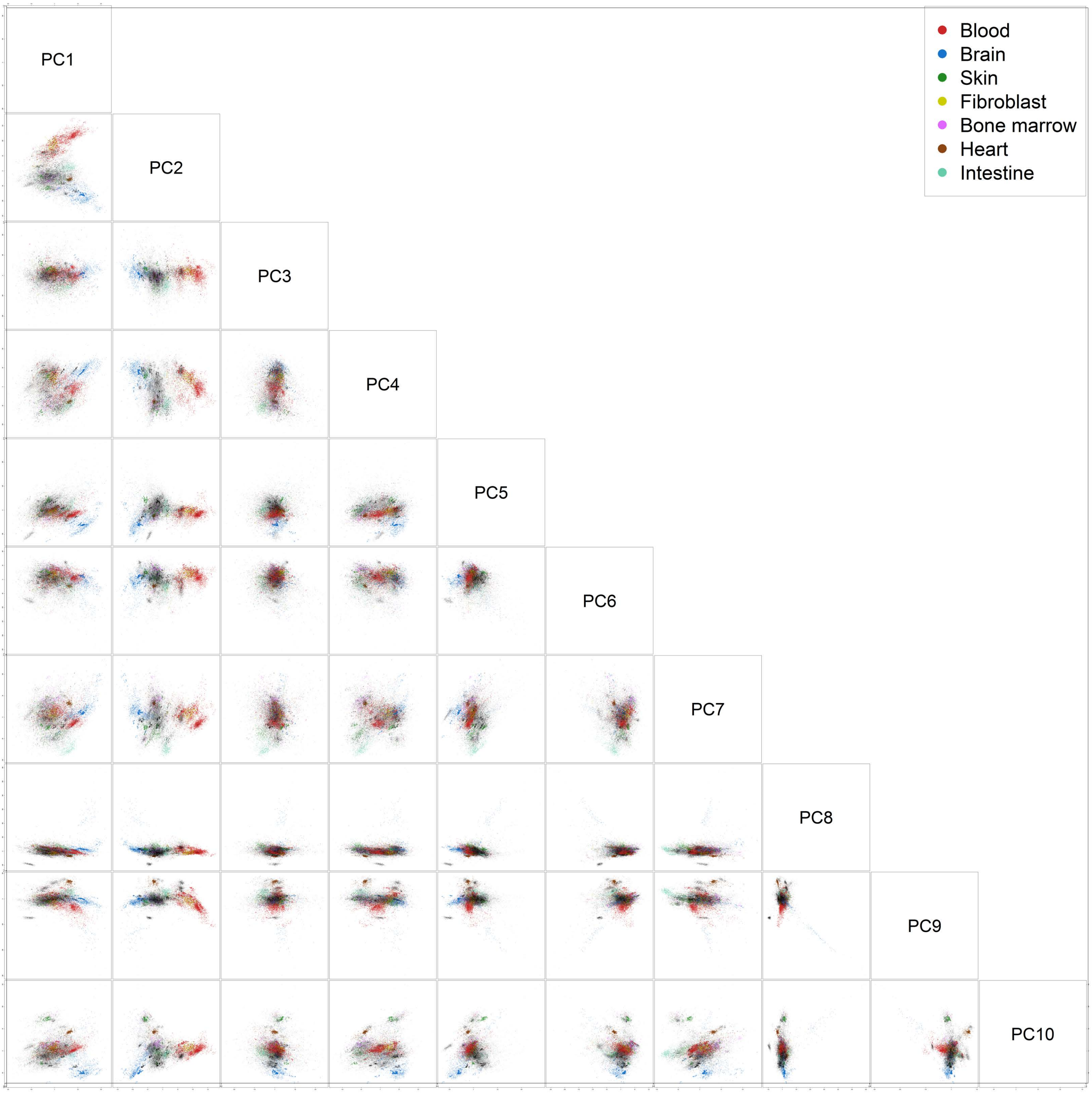
PC1
PC2
PC3
PC4
PC5
PC6
PC7
PC8
PC9
PC10
Blood
Brain
Skin
Fibroblast
Bone marrow
Heart
Intestine

Supplementary figure 16: Outlier samples in PC sample scores of PC 8 and PC 9.

Inspection of the first 10 PCs revealed outliers on PC 8 and PC 9. We set a cutoff at PC 8 > 100 and PC 9 < -50 to select the outlier samples and retrieved the tissue/cell-type annotation for these samples. Among the outlier samples, 14 were testis samples originating from five different studies and 60 were brain samples of which most are annotated with cancer. Additionally, a number of other outlier samples were observed, and some of these were also cancer samples. A number of studies support that olfactory genes are expressed in the testes [20,21]. The fact that the glioblastoma samples were also outliers in this PC could be the result of accidental activation of these genes by the glioblastoma. Based on this observation, we concluded the outlier signal is of biological nature and decided to keep this in the data rather than removing it.

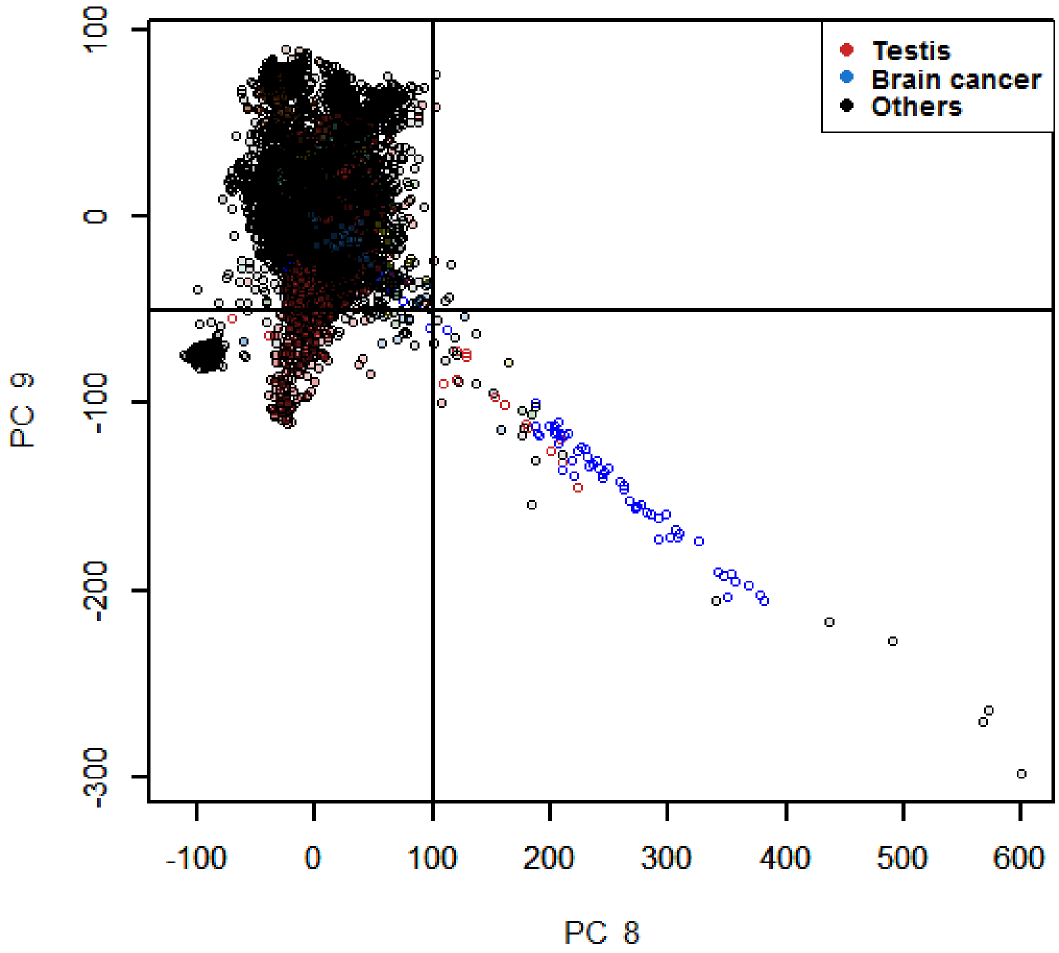

Supplementary tables

Supplementary table 1: A list of 83 diagnosed patients with Mendelian disorders and corresponding predictions with GADO.

The phenotype of the patients was described with HPO terms best matching their phenotypes. These patients were originally diagnosed through exome sequencing with analysis of a gene panel or the entire exome.

The rank of the causative gene in the GADO predictions was determined using the corresponding HPO terms and the genes GAVIN flagged as harboring a potentially causative variant for each patient respectively.

**Sample**	**Gene**	**HPO terms**	**GADO Rank**	**Total disease genes with gavin variants**
DiagnosedPatient1	TTN	HP:0001644	1	59
DiagnosedPatient2	TTN	HP:0001644	1	49
DiagnosedPatient3	MYBPC3	HP:0001644	1	40
DiagnosedPatient4	MYH7	HP:0001644	1	64
DiagnosedPatient5	MYH7	HP:0005157	1	59
DiagnosedPatient6	MYL2	HP:0001644	1	47
DiagnosedPatient7	CYB561	HP:0001278	1	43
DiagnosedPatient8	RBM10	HP:0001883<br>HP:0000609<br>HP:0012736	1	51
DiagnosedPatient9	TTN	HP:0001644	1	50
DiagnosedPatient10	MYL2	HP:0001644<br>HP:0004764	1	46
DiagnosedPatient11	MYH7	HP:0001644<br>HP:0000822	1	47
DiagnosedPatient12	MYL2	HP:0001644<br>HP:0001942	1	56
DiagnosedPatient13	MYH7	HP:0001644<br>HP:0012817	1	62

DiagnosedPatient14	MYH7	HP:0001644<br>HP:0012817	1	59
DiagnosedPatient15	MYL2	HP:0001644<br>HP:0004755	1	48
DiagnosedPatient16	USP9X	HP:0000707<br>HP:0000453<br>HP:0002023<br>HP:0100259	1	57
DiagnosedPatient17	SPG7	HP:0001258	1	47
DiagnosedPatient18	BBS5	HP:0000548<br>HP:0001513	1	56
DiagnosedPatient19	SEPN1	HP:0003011	1	50
DiagnosedPatient20	DDX3X	HP:0000707	1	43
DiagnosedPatient21	TTN	HP:0001644	2	52
DiagnosedPatient22	TNNT2	HP:0001644	2	63
DiagnosedPatient23	MYL2	HP:0005157	2	58
DiagnosedPatient24	PDE6B	HP:0000510	2	51
DiagnosedPatient25	DYNC1H1	HP:0000478<br>HP:0011343<br>HP:0005484<br>HP:0000565	2	92
DiagnosedPatient26	EHMT1	HP:0000271<br>HP:0011750<br>HP:0001249	2	61
DiagnosedPatient27	USH2A	HP:0000510	2	60
DiagnosedPatient28	AFG3L2	HP:0001251<br>HP:0002066<br>HP:0002470<br>HP:0007240<br>HP:0002131	3	40
DiagnosedPatient29	SPEG	HP:0001644<br>HP:0003198	3	43
DiagnosedPatient30	SCN5A	HP:0011701<br>HP:0001644<br>HP:0004755	3	61

DiagnosedPatient31	TTN	HP:0001644	3	47
DiagnosedPatient32	SPG7	HP:0002313	3	54
DiagnosedPatient33	RPGR	HP:0000510	3	45
DiagnosedPatient34	SLC12A7 TECTB GJB3	HP:0000407	3	45
DiagnosedPatient35	PLD3	HP:0001251 HP:0002066 HP:0002470 HP:0007240 HP:0002131	4	42
DiagnosedPatient36	RPE65	HP:0007875	4	62
DiagnosedPatient37	NDUFS7	HP:0000707	4	55
DiagnosedPatient38	CASK	HP:0003011 HP:0000271	4	49
DiagnosedPatient39	PSTPIP1	HP:0001817 HP:0001911 HP:0011034	4	70
DiagnosedPatient40	HSPG2	HP:0002486 HP:0011338 HP:0001638	4	58
DiagnosedPatient41	RPE65	HP:0000510	4	58
DiagnosedPatient42	ALPK3	HP:0001644	5	49
DiagnosedPatient43	ARID1B	HP:0000271 HP:0000707 HP:0002086	5	57
DiagnosedPatient44	GJB2	HP:0008527	6	61
DiagnosedPatient45	KAT6B	HP:0000707	7	55
DiagnosedPatient46	NEK1	HP:0000478	7	57
DiagnosedPatient47	NPC1	HP:0000707	7	53
DiagnosedPatient48	PDHA1	HP:0001939 HP:0001626 HP:0002086	8	67
DiagnosedPatient49	MAGEL2	HP:0100704 HP:0001763	8	43

		HP:0000494<br>HP:0000047		
DiagnosedPatient50	MTM1	HP:0001319	9	47
DiagnosedPatient51	KCNT1	HP:0002133<br>HP:0000707<br>HP:0001638	9	49
DiagnosedPatient52	PIK3R2	HP:0030680<br>HP:0000256<br>HP:0002126	10	47
DiagnosedPatient53	RARS	HP:0000929	10	44
DiagnosedPatient54	PIEZO2	HP:0000924	10	50
DiagnosedPatient55	TTN SLC37A4	HP:0001644<br>HP:0001882<br>HP:0002037<br>HP:0031123<br>HP:0001987	10.5	58
DiagnosedPatient56	MEGF10	HP:0001319	12	58
DiagnosedPatient57	KANSL1	HP:0000750<br>HP:0000717	12	54
DiagnosedPatient58	RAPSN	HP:0000271<br>HP:0003808<br>HP:0001324	13	57
DiagnosedPatient59	GJB2	HP:0008527	14	76
DiagnosedPatient60	SOD2	HP:0001644	15	39
DiagnosedPatient61	TBCK	HP:0001319<br>HP:0012727<br>HP:0000271	15	60
DiagnosedPatient62	GLB1	HP:0001644	18	88
DiagnosedPatient63	STXBP1	HP:0000707	19	60
DiagnosedPatient64	PRKCG	HP:0001251<br>HP:0002066<br>HP:0002470<br>HP:0007240<br>HP:0002131	21	52

DiagnosedPatient65	FAT1	HP:0001251<br>HP:0002066<br>HP:0002470<br>HP:0007240<br>HP:0002131	21	60
DiagnosedPatient66	PTPN11	HP:0000474<br>HP:0000368<br>HP:0006610<br>HP:0001939	22	63
DiagnosedPatient67	SPG7	HP:0002062<br>HP:0000729	22	54
DiagnosedPatient68	GFER	HP:0003128<br>HP:0001943<br>HP:0001319<br>HP:0002093	23	65
DiagnosedPatient69	KCNQ2	HP:0002197<br>HP:0002133<br>HP:0000707<br>HP:0001939	23	52
DiagnosedPatient70	USP9X	HP:0001626	23	64
DiagnosedPatient71	CACNA1A	HP:0001251<br>HP:0002066<br>HP:0002470<br>HP:0007240<br>HP:0002131	24	59
DiagnosedPatient72	CHD7	HP:0010880<br>HP:0012020<br>HP:0001789<br>HP:0000271<br>HP:0001939<br>HP:0003011	25	65
DiagnosedPatient73	TMEM240	HP:0001251<br>HP:0002066<br>HP:0002470	26	54

		HP:0007240<br>HP:0002131		
DiagnosedPatient74	GRIN2B	HP:0001249<br>HP:0003019<br>HP:0003011<br>HP:0000707	26	55
DiagnosedPatient75	DNMT3A	HP:0000478	27	46
DiagnosedPatient76	PDE6B	HP:0000478	28	48
DiagnosedPatient77	PAX6	HP:0000707<br>HP:0001249	30	62
DiagnosedPatient78	FAT2	HP:0001251<br>HP:0002066<br>HP:0002470<br>HP:0007240<br>HP:0002131	32	45
DiagnosedPatient79	MAP3K7	HP:0009099<br>HP:0001193<br>HP:0005656<br>HP:0004209	32	60
DiagnosedPatient80	KBTBD13	HP:0000271<br>HP:0009602<br>HP:0001319	36	58
DiagnosedPatient81	RYR1	HP:0003793	37	59
DiagnosedPatient82	GJB2	HP:0008619	40	62
DiagnosedPatient83	CRLF1	HP:0002015<br>HP:0006610<br>HP:0003186<br>HP:0000707<br>HP:0025031	49	83

Supplementary table 2: Comparison between GADO and Exomiser predictions using a list of 83 diagnosed patients with Mendelian disorders.

Similar to the analysis with GADO, Exomiser [22] was used to predict causative genes in the 83 solved samples. The Exomiser gene files, separated by different inheritance modes, were concatenated and the rank of the causative gene was determined. If a gene was present in multiple output files, the highest (best) rank was used. When genes were scored equally, the average rank of all genes with equal scores was reported. When multiple causative genes were annotated for a patient, the median rank of each was determined and is reported in the table. We also list the rank of GADO with and without incorporating existing knowledge when ranking the genes with variants selected by Exomiser.

Case	Causative gene	Number genes selected by Exomiser	Exomiser rank	GADO rank without existing knowledge	GADO rank including existing knowledge
DiagnosedPatient1	TTN	901	1	2	2
DiagnosedPatient10	MYL2	714	1	1	1
DiagnosedPatient12	MYL2	639	1	1	1
DiagnosedPatient13	MYH7	766	1	1	1
DiagnosedPatient14	MYH7	793	1	1	1
DiagnosedPatient17	SPG7	610	1	12	6
DiagnosedPatient2	TTN	849	1	2	2
DiagnosedPatient21	TTN	713	1	1	1
DiagnosedPatient23	MYL2	681	1	1	1
DiagnosedPatient27	USH2A	565	1	2	2
DiagnosedPatient28	AFG3L2	650	1	19	8
DiagnosedPatient30	SCN5A	615	1	4	4
DiagnosedPatient4	MYH7	717	1	1	1
DiagnosedPatient41	RPE65	548	1	13	7
DiagnosedPatient44	GJB2	578	1	62	12
DiagnosedPatient5	MYH7	872	1	7	7
DiagnosedPatient56	MEGF10	595	1	52	8
DiagnosedPatient60	SOD2	646	1	113	114
DiagnosedPatient62	GLB1	961	1	114	18
DiagnosedPatient64	PRKCG	552	1	213	18

DiagnosedPatient71	CACNA1A	633	1	188	83
DiagnosedPatient73	TMEM240	655	1	188	14
DiagnosedPatient32	SPG7	540	2	4	4
DiagnosedPatient42	ALPK3	989	2	18	18
DiagnosedPatient59	GJB2	663	2	83	21
DiagnosedPatient22	TNNT2	676	3	4	4
DiagnosedPatient29	SPEG	638	3	11	11
DiagnosedPatient36	RPE65	549	3	12	9
DiagnosedPatient34	GJB3<br>TECTB<br>SLC12A7	536	4	18	18
DiagnosedPatient67	SPG7	588	4	231	114
DiagnosedPatient11	MYH7	842	5	3	3
DiagnosedPatient43	ARID1B	592	5	23	9
DiagnosedPatient55	TTN<br>SLC37A4	670	5	222.5	195
DiagnosedPatient18	BBS5	531	7	13	11
DiagnosedPatient77	PAX6	554	13	197	11
DiagnosedPatient45	KAT6B	542	17	26	43
DiagnosedPatient25	DYNC1H1	688	21	3	5
DiagnosedPatient69	KCNQ2	715	22	212	13
DiagnosedPatient49	MAGEL2	575	24	43	48
DiagnosedPatient80	KBTBD13	526	25	298	306
DiagnosedPatient72	CHD7	965	27	262	7
DiagnosedPatient76	PDE6B	499	31	175	29
DiagnosedPatient79	MAP3K7	779	32	233	33
DiagnosedPatient7	CYB561	967	35	38	43
DiagnosedPatient19	SELENON	575	40	221	40
DiagnosedPatient39	PSTPIP1	586	40	19	20
DiagnosedPatient74	GRIN2B	542	41	225	38
DiagnosedPatient66	PTPN11	794	47	226	1
DiagnosedPatient51	KCNT1	911	48	59	19
DiagnosedPatient40	HSPG2	544	55	14	9
DiagnosedPatient75	DNMT3A	521	59	217	223

DiagnosedPatient83	CRLF1	914	76	437	130
DiagnosedPatient26	EHMT1	526	82	6	7
DiagnosedPatient38	CASK	540	85	23	20
DiagnosedPatient20	DDX3X	527	89	5	5
DiagnosedPatient70	USP9X	572	89	140	23
DiagnosedPatient8	RBM10	540	89	1	1
DiagnosedPatient33	RPGR	537	91	7	7
DiagnosedPatient50	MTM1	884	111	91	91
DiagnosedPatient48	PDHA1	1004	141	26	43
DiagnosedPatient68	GFER	755	141	97	103
DiagnosedPatient35	PLD3	585	152	15	22
DiagnosedPatient6	MYL2	664	158	1	1
DiagnosedPatient47	NPC1	545	171	36	34
DiagnosedPatient53	RARS	548	179	88	24
DiagnosedPatient65	FAT1	658	192	186	192
DiagnosedPatient24	PDE6B	577	202	20	7
DiagnosedPatient37	NDUFS7	547	218	8	8
DiagnosedPatient57	KANSL1	489	231	86	32
DiagnosedPatient61	TBCK	922	252	207	54
DiagnosedPatient54	PIEZO2	519	299	79	26
DiagnosedPatient78	FAT2	564	314	355	357
DiagnosedPatient15	MYL2	760	Not Reported	1	1
DiagnosedPatient16	USP9X	570	Not Reported	2	1
DiagnosedPatient3	MYBPC3	669	Not Reported	1	1
DiagnosedPatient31	TTN	640	Not Reported	2	2
DiagnosedPatient46	NEK1	573	Not Reported	20	56
DiagnosedPatient52	PIK3R4	561	Not Reported	51	15

DiagnosedPatient58	RAPSN	782	Not Reported	134	1
DiagnosedPatient63	STXBP1	554	Not Reported	98	33
DiagnosedPatient81	RYR1	586	Not Reported	216	216
DiagnosedPatient82	GJB2	617	Not Reported	297	297
DiagnosedPatient9	TTN	644	Not Reported	1	1

Supplementary table 3: A list of 61 undiagnosed patients with suspected Mendelian disorders.

Our patients were described with HPO terms best matching their phenotypes. We aimed to use terms that are as specific as possible, thus aiming to avoid HPO terms that describe a broader, less-specific phenotype.

**Annonemized**	**HPO terms**	**Number of genes prioritization Z-score ≥ 5**
Case 1	HP:0001644	2
Case 2	HP:0001644	5
Case 3	HP:0001638 HP:0001701	3
Case 4	HP:0001644	5
Case 5	HP:0001644	4
Case 6	HP:0001644 HP:0001636	6
Case 7	HP:0001644 HP:0011675	2
Case 8	HP:0001644 HP:0001250	2
Case 9	HP:0001644 HP:0004755	4
Case 10	HP:0001644 HP:0001712 HP:0001250	1
Case 11	HP:0001644	3
Case 12	HP:0001644	2
Case 13	HP:0001644	1
Family 1	HP:0001644	1
Family 2	HP:0001644	1
Family 3	HP:0001644	3
Family 4	HP:0001644	2
Family 5	HP:0001644	0
Family 6	HP:0001644	1
Family 7	HP:0001638	1
Family 8	HP:0001644	1
Family 9	HP:0001644 HP:0005110 HP:0031546 HP:0006704	2
Family 10	HP:0001644	0
Family 11	HP:0001644	2

Family 12	HP:0001638	1
Family 13	HP:0001644	4
Family 14	HP:0001638	3
Case 14	HP:0001644	2
Case 15	HP:0001638	4
Case 16	HP:0001263 HP:0001249 HP:0000717 HP:0002300 HP:0002360 HP:0000664	12
Case 17	HP:0001249 HP:0004322 HP:0000252	7
Case 18	HP:0001249 HP:0000729 HP:0002300	8
Case 19	HP:0008066 HP:0008064	9
Case 20	HP:0040194 HP:0000707	2
Case 21	HP:0000098 HP:0000707	1
Case 22	HP:0003458 HP:0003715 HP:0003789	2
Case 23	HP:0001522	0
Case 24	HP:0001305 HP:0001263	1
Case 25	HP:0012302	2
Case 26	HP:0002092 HP:0030875	2
Case 27	HP:0002197	1
Case 28	HP:0000364 HP:0000707	2
Case 29	HP:0000252 HP:0002092	1
Case 30	HP:0002791	0
Case 31	HP:0002197	1
Case 32	HP:0001641	0
Case 33	HP:0030968 HP:0001928	0
Case 34	HP:0100495 HP:0011675 HP:0001699	3
Case 35	HP:0007402 HP:0100022 HP:0011400 HP:0011344 HP:0002375	17

Case 36	HP:0001684 HP:0002905 HP:0011682 HP:0004383	6
Case 37	HP:0004322 HP:0001249	10
Case 38	HP:0001263 HP:0000506	0
Case 39	HP:0002123	1
Case 40	HP:0004481 HP:0011342	2
Case 41	HP:0001319	0
Case 42	HP:0002791 HP:0001520 HP:0001270	0
Case 43	HP:0001789	0
Case 44	HP:0011675 HP:0001714	6
Case 45	HP:0011107	0
Case 46	HP:0003493 HP:0002583	6
Case 47	HP:0012649 HP:0002583 HP:0001890	8

Supplementary References

1. Silvester, N. *et al.* Content discovery and retrieval services at the European Nucleotide Archive. *Nucleic Acids Res.* **43**, D23–D29 (2015).

2. Lachmann, A. *et al.* Massive mining of publicly available RNA-seq data from human and mouse. doi:10.1038/s41467-018-03751-6

3. Deelen, P. *et al.* Calling genotypes from public RNA-sequencing data enables identification of genetic variants that affect gene-expression levels. *Genome Med.* **7**, 30 (2015).

4. Cunningham, F. *et al.* Ensembl 2015. *Nucleic Acids Res.* **43**, D662–D669 (2015).

5. Bray, N. L., Pimentel, H., Melsted, P. & Pachter, L. Near-optimal probabilistic RNA-seq quantification. *Nat. Biotechnol.* **34**, 525–527 (2016).

6. Anders, S. & Huber, W. Differential expression analysis for sequence count data. *Genome Biol.* **11**, R106 (2010).

7. Vinogradov, A. E. DNA helix: the importance of being GC-rich. *Nucleic Acids Res.* **31**, 1838–44 (2003).

8. Cronbach, L. J. Coefficient alpha and the internal structure of tests. *Psychometrika* **16**, 297–334 (1951).

9. Bresciani, M. J. *et al.* Examining Design and Inter-Rater Reliability of a Rubric Measuring Research Quality across Multiple Disciplines - Practical Assessment, Research & Evaluation. **14**, (2009).

10. Fehrmann, R. S. N. *et al.* Gene expression analysis identifies global gene dosage sensitivity in cancer. *Nat. Genet.* **47**, 115–125 (2015).

11. Van Der Maaten, L. & Hinton, G. Visualizing Data using t-SNE. *J. Mach. Learn. Res.* **9**,

2579–2605 (2008).

12. Krijthe, J. H. T-Distributed Stochastic Neighbor Embedding using Barnes-Hut. *T-Distributed Stochastic Neighbor Embedding using Barnes-Hut* (2015).

13. Tranchevent, L.-C. *et al.* Candidate gene prioritization with Endeavour. *Nucleic Acids Res.* **44**, W117–W121 (2016).

14. Chen, J., Bardes, E. E., Aronow, B. J. & Jegga, A. G. ToppGene Suite for gene list enrichment analysis and candidate gene prioritization. *Nucleic Acids Res.* **37**, W305–W311 (2009).

15. Frey, B. J. & Dueck, D. Clustering by passing messages between data points. *Science* **315**, 972–6 (2007).

16. Harakalova, M. *et al.* A systematic analysis of genetic dilated cardiomyopathy reveals numerous ubiquitously expressed and muscle-specific genes. *Eur. J. Heart Fail.* **17**, 484–493 (2015).

17. Herkert, J. C. *et al.* Toward an effective exome-based genetic testing strategy in pediatric dilated cardiomyopathy. *Genet. Med.* (2018). doi:10.1038/gim.2018.9

18. Zhang, B. & Horvath, S. A General Framework for Weighted Gene Co-Expression Network Analysis. *Stat. Appl. Genet. Mol. Biol.* **4**, Article17 (2005).

19. Dolgin, E. The most popular genes in the human genome. *Nature* **551**, 427–431 (2017).

20. Fukuda, N. & Touhara, K. Developmental expression patterns of testicular olfactory receptor genes during mouse spermatogenesis. *Genes to Cells* **11**, 71–81 (2005).

21. Goto, T., Salpekar, A. & Monk, M. Expression of a testis-specific member of the olfactory receptor gene family in human primordial germ cells. *Mol. Hum. Reprod.* **7**,

553–8 (2001).

22. Smedley, D. *et al.* Next-generation diagnostics and disease-gene discovery with the Exomiser. *Nat. Protoc.* **10**, 2004–15 (2015).
