# Supplementary material for: Improving the diagnostic yield of exome- sequencing by predicting gene–phenotype associations using large-scale gene expression analysis
Source: Nat Commun. 2019 Jun 28;10:2837. doi: 10.1038/s41467-019-10649-4 (PMC6599066; doi:10.1038/s41467-019-10649-4)
Supplement: Supplementary file 6 — Description of Additional Supplementary Files [file 41467_2019_10649_MOESM6_ESM.docx]

**Title:** Supplementary data 1
**Description:** A list of samples annotated in the European Nucleotide Archive June 30, 2016. Samples that are download from the ENA database. The total number of reads assessed and mapped, and the percentage of reads mapped, are based on Kallisto summary reports.

**Title:** Supplementary data 2
**Description:** OMIM disease gene relations, prioritization Z-score and predictability scores. Per OMIM disease we report the prioritization Z-scores of the associated genes. For each gene we also list the gene predictability score.

**Title:** Supplementary data 3
**Description:** Candidate genes for solved and unsolved cases. Lists of candidate genes identified in each patient, using GAVIN, that are used for benchmarking.

**Title:** Supplementary data 4
**Description:** Prioritization performance comparison in unsolved cases. For the unsolved cases for which GADO identified a strong candidate gene we tested the performance of Exomiser, ENDEAVOR and ToppGene to show how GADO is complementary to existing tools to identify novel disease genes
